# An email-based intervention to improve the number and timeliness of letters sent from the hospital outpatient clinic to the general practitioner: A pair-randomized controlled trial

**DOI:** 10.1371/journal.pone.0185812

**Published:** 2017-10-23

**Authors:** Stephanie Medlock, Juliette L. Parlevliet, Danielle Sent, Saeid Eslami, Marjan Askari, Derk L. Arts, Joost B. Hoekstra, Sophia E. de Rooij, Ameen Abu-Hanna

**Affiliations:** 1 Department of Medical Informatics, Academic Medical Center, University of Amsterdam, Amsterdam Public Health Research Institute, Amsterdam, the Netherlands; 2 Department of Internal Medicine, Academic Medical Center, University of Amsterdam, Amsterdam, the Netherlands; 3 Pharmaceutical Research Center, School of Pharmacy, Mashhad University of Medical Sciences, Mashhad, Iran; 4 Department of Information and Computing Sciences, Universiteit Utrecht, Utrecht, The Netherlands; 5 Department of Internal Medicine, St Antonius Hospital, Nieuwegein, the Netherlands; 6 University Center of Geriatric Medicine, University of Groningen, Groningen, The Netherlands; University of Alabama at Birmingham School of Health Professions, UNITED STATES

## Abstract

**Objective:**

Letters from the hospital to the general practitioner are important for maintaining continuity of care. Although doctors feel letters are important, they are often not written on time. To improve the number and timeliness of letters sent from the hospital outpatient department to the general practitioner using an email-based intervention evaluated in a randomized controlled trial.

**Materials and methods:**

Users were interviewed to determine the requirements for the intervention. Due to high between-doctor variation at baseline, doctors were matched for baseline performance and pair-randomized. The effectiveness of the intervention was assessed using meta-analytic methods. The primary outcome was the number of patient visits which should have generated a letter that had a letter by 90 days after the visit. Satisfaction was assessed with an anonymous survey.

**Results:**

The intervention consisted of a monthly email reminder for each doctor containing a list of his or her patients who were (over)due for a letter. Doctors in the intervention group had 21% fewer patient visits which did not have a letter by 90 days (OR = 5.7, p = 0.0020). Satisfaction with the system was very high.

**Discussion:**

This study examines the effect of a simple reminder in absence of other interventions, and provides an example of an effective non-interruptive decision support intervention.

**Conclusion:**

A simple email reminder improved the number and timeliness of letters from the outpatient department to the general practitioner, and was viewed as a useful service by its users.

## Introduction

Letters from the hospital doctor to the general practitioner (GP) are the main channel of communication between the hospital and the primary care provider [1]. In the Netherlands, the GP is the central coordinator for the patient’s care [2], and thus it is important that the GP remains informed about the care the patient receives at the hospital, whether as an inpatient or outpatient. Poor communication between the hospital and the GP at discharge has been associated with higher rates of readmission [3], and clinicians recognize good communication between the hospital and the GP as an important component of patient safety [4]. However, in spite of this recognition, letters to the GP are often delayed or forgotten [5].

Computerized clinical decision support can be defined as any computerized system which helps in making clinical decisions [6], including assistance with communication and documentation tasks [7,8]. Automated reminders have been shown to be effective in supporting clinical documentation tasks [9,10], but thus far have not been applied to help doctors identify patients for whom letters need to be written and ensure that the letters are sent in a timely manner. Although alerts which are interruptive [11] and require a reason for dismissal [12] have been shown to be more effective than alerts without these characteristics, interruption also has the potential to negatively impact patient care [13]. Thus, the objective of this study was to develop and evaluate a non-interruptive computerized clinical decision support intervention to improve the number and timeliness of letters sent to the GP from the hospital's outpatient clinic.

## Methods

The Medical Ethics Committee at our institution reviewed the plan for this study and determined it was exempt from the need for ethics committee review.

### Setting

The study took place in a tertiary-care, university medical center seeing 56,000 patients per year [14]. The general internal medicine and geriatric medicine outpatient clinics agreed to participate in the trial. Together, these clinics are staffed by 11 staff doctors and 5 to 7 residents, and see approximately 4000 patients per year.

### Intervention design

We planned to develop and perform a patient-specific, email-based decision support intervention. To determine the content, format, timing, and interaction options for the intervention, we conducted semi-structured interviews consisting of three questions pertaining to the following aspects: the doctor’s current workflow regarding letters, the parameters they would like (e.g. how often they wanted reminders), and what capabilities and style of user interface they would like for controlling the content of the emails. Interview subjects were chosen by purposive sampling to represent both staff doctors and residents in both departments. Based on these results, we developed a stand-alone, open-source computer program to generate email reminders. The query to match letters to patients was refined for each participating department to minimize the number of false positive reminders (i.e. a patient visit is included in the email, but a letter was already written for that visit) and the number of false negatives (i.e. a letter is incorrectly attributed to a patient visit, thus no reminder is generated).

### Baseline and trial design

The trial was registered with the Netherlands Trial Registry (NTR3369). Due to high between-doctor variation in baseline performance, pairwise randomization was used for the randomized controlled trial. Baseline performance was measured as the percentage of patient visits to the outpatient department in the 12 months prior to the start of the study which did not have a letter associated with the visit by 90 days after the visit. A cut-off of 90 days was chosen because, in the baseline data set, fewer than 10% of letters that were not written by 90 days after the visit were ever written at all. Pairs of doctors were assigned to one another to minimize the within-pair difference in this outcome measure, and one member of each pair was randomly selected to be in the intervention group. Randomization was performed by SM using the random integer generator from the website https://www.random.org, by first creating the pairs and then assigning each pair a 0 (first doctor in control group) or 1 (first doctor in intervention group). Doctors consisted of both staff doctors and residents. Residents move to different departments during their training, and the new resident inherits the outpatients of his predecessor. Therefore, new doctors entering the service also kept the same pair assignment as their predecessor, allowing patients to remain in the same arm of the trial. For the purposes of analysis, the new combination of doctors was considered a new pair. For example, if Dr. A and Dr. B are a pair, and Dr. B leaves and is replaced by Dr. C, then Dr. A and Dr. C form a new pair. Thus, the same doctor could be a member of more than one pair during the trial, but only one pair during any given time period. A doctor was required to stay on the outpatient clinic for at least two consecutive months during the trial to be entered into the analysis. Based on this design, we estimated that we would need to continue the trial for 6 to 12 months to include enough doctors in the analysis.

### Analysis of trial data

The main outcome measure was the percentage of patients who visited the outpatient clinic during the trial who had a letter written within 90 days of their visit. To analyze the clustered, pair-randomized data, we chose a meta-analytic approach as suggested by Thompson et al. [15]. The data from each pair of doctors is analyzed as if it were a trial in the meta-analysis. We expected variability in the true effect, as the intervention could reasonably be expected to affect some doctors more than others. Therefore a mixed-effects model was used, using a random-effects model with the Restricted Maximum Likelihood (REML) estimator and the difference in performance within each pair at the time of randomization as a moderator variable (R version 3.1.0, package metafor) [16,17].

As a secondary analysis, we repeated the meta-analysis with the patient as the unit of analysis rather than the patient visit. Patients can visit more than once during the study period, and may see different doctors. Each visit has the potential to trigger a reminder, and thus is the unit where the intervention has its main effect. However, in terms of clinical relevance, it is more important to ask whether the patient has a letter associated with their visit. Thus, we also performed the analysis with the patient as the unit of analysis rather than the patient-visit. For this analysis, patients were assigned to the doctor that they saw on their first visit during the trial. We also compared the before-after performance of both the control and intervention groups using a chi-squared test. The median number of days between the visit date and the start date of the letter and the median number of days between the visit date and the date the letter was sent to the GP was measured in the control and intervention groups and compared using the Mann-Whitney U test. In addition, we performed a post-hoc sensitivity analysis with the natural logarithm of the ratio of the number of patients seen by the control doctor to the number of patients seen by the intervention doctor as an additional moderator variable to control for workload.

**Fig 1:**
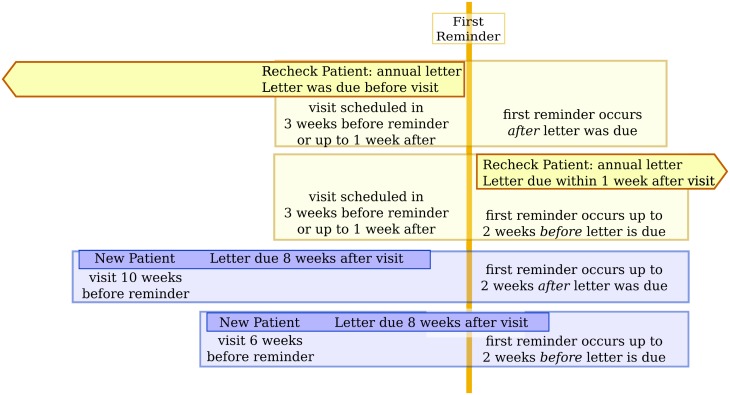
Range of possible relationships between the patient visit, the letter due date, and the first reminder. Recheck patients should get a letter every year (or 2 years for internal medicine). If a recheck patient had a visit during the trial, they were eligible for a reminder if the letter was due before the visit (sometimes, long before the start of the trial) or up to 1 week after the visit. Reminders were sent for appointments that had already occurred and appointments scheduled for the coming week, meaning that reminders were sent no more than 2 weeks before the letter was due. New patients should get a letter within 8 weeks of their visit. Patients were considered eligible for a reminder if they did not yet have a letter at 6 weeks after their visit, meaning that the first reminder for a patient was issued 6 to 10 weeks (an average of 8 weeks) after the visit.

The software was written in Java as open source software. It gathers a list of patients with a recent or planned visit to the outpatient clinic, checks whether the patient has a letter that matches to the visit (according to the criteria set for each department), and if not, marks the patient as eligible for a reminder. The software checks the agenda, checks for letters, and then sends reminders, so all information is up to date when the reminders are sent. Timelines of the possible relationships between the visit date and the reminder date are illustrated in Fig 1.

### User satisfaction survey

To assess the users’ perception of the system, two of the researchers (SM and DS) constructed a survey based on the Information Systems Success Model (DeLone/McLean model) [18]. The constructs from this model that were relevant to this intervention were identified and instantiated with questions from existing standard questionnaires: the IBM Computer System Usability Questionnaire [19], the System Usability Scale [20], and the Computer User Satisfaction questionnaire [21]. The resulting survey (see S1 File) was assessed for face validity and completeness by two experts in medical informatics (SE and AA). It was distributed to the doctors in the intervention arm of the trial as an email to their hospital email address. A paper version was also distributed in the hospital mail boxes for doctors who were still working in our hospital. Surveys returned in paper form were anonymous.

## Results

### Intervention design

**Fig 2:**
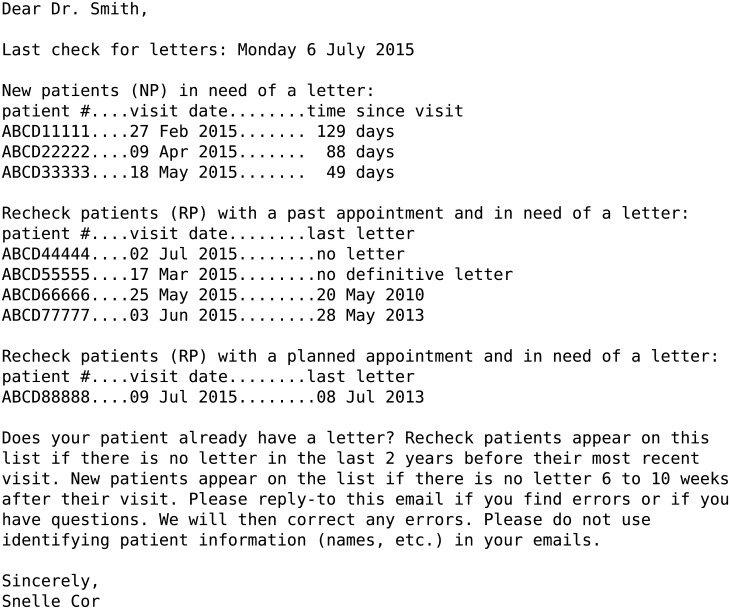
Example of an email reminder.

We conducted interviews with a purposive sample of five doctors (the heads of the geriatrics and internal medicine departments, one additional senior doctor, and one resident from each department). Based on these interviews, we designed an intervention consisting of a monthly, plain-text email formatted in three sections (Fig 2). Hospital policy states that letters should be sent for “new patients” (the patient’s first visit to the department) within 2 months of the visit. The policy for recheck patients varies per department; the departments in our study recommend a letter every 2 years (internal medicine) or every year (geriatrics). Because the email was sent monthly, reminders would be sent for new patients 6 to 10 weeks after their visit. If any of the sections had no patients due for letters, that section contained a message that said “Well done! No (new/recheck) patients are in need of letters!” The subject line of the email read “letters due” and was the same for emails with or without patients in need of letters. The emails were signed “Snelle Cor” (“Cor” alludes to correspondence and is a common Dutch first name, and “snelle” means fast). The doctors we interviewed did not want a user interface for controlling the content of the reminders, therefore we instead added information about contacting the administrator to the emails.

### Baseline

In the year before the trial there were 8173 patient visits in the internal medicine and geriatrics services of the outpatient clinic, with an average of 2.4 visits per patient (3473 patients). A total of 9.3% of the 8173 visits were >90 days overdue for a letter, 9.4% in the control group and 9.1% in the intervention group (**χ**^2^ = 0.25, p = 0.62). This percentage varied between doctors, ranging from 0.3% to 27.5%. Doctors were paired to minimize the difference in initial performance between the intervention and control group; the median absolute difference in the baseline between members of a pair was 1.0% (IQR 0.3–2.2%).

### Analysis of trial data

#### Participants

**Fig 3:**
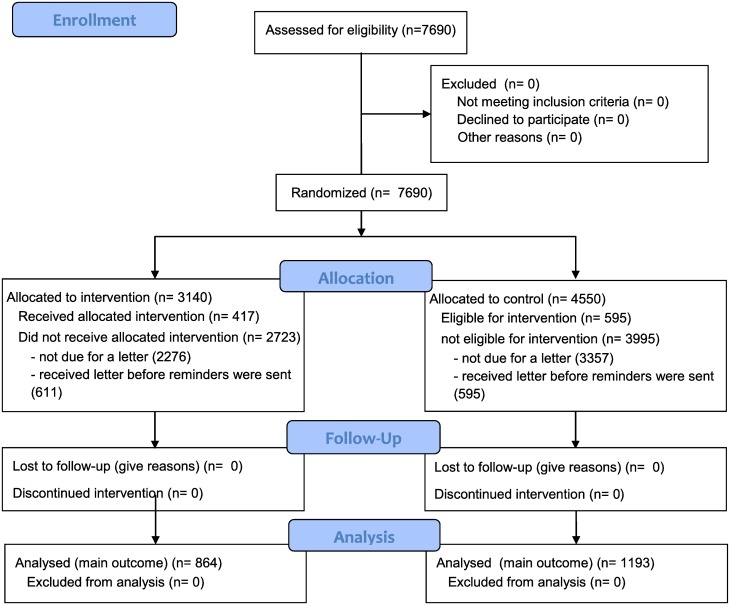
CONSORT diagram. All 7690 patients visiting the internal medicine or geriatrics outpatient clinic were included. Doctors allocated to the intervention group received reminders for patients who were due for a letter during the trial.

The trial included patients visiting the internal medicine or geriatrics service of the outpatient clinic between 1 April 2012 and 31 March 2013. The intervention was preceded by an announcement at the monthly staff meeting of the included departments. The trial included 26 doctors, assigned to 15 pairs (as described in the Methods, if one doctor in the pair left the service, a new doctor would take over the patients of the one who left, and also be given the same randomization assignment). The trial started with 16 doctors in 8 pairs; of these, 6 doctors remained on the service for the whole trial period. The pairs lasted a median of 6 months (range 2 to 12 months). A total of 7690 patient visits were included in the trial, with an average of 2.3 visits per patient (3310 patients). Participating doctors saw a median of 27 patients/month (2 new patients and 26 recheck patients; range 2–112, IQR 15–65), with a median of 7 patients needing a letter in any one month (range 0–48, IQR 5–15). The patient-visits consisted of 7253 recheck visits (3064 patients) and 437 new patient visits (434 patients). A total of 2057 of these visits should have been followed by a letter (864/3140 intervention, 27.5%; 1193/4550 control, 26.2%), and thus were eligible for inclusion in the main analysis (Fig 3). Of these, 1206 (611 intervention, 71%; 595 control, 50%) received a letter within 90 days of their visit. For 981 of those visits (417 intervention, 564 control), the letter had not yet been written at the time the monthly reminders were sent, and thus would generate a reminder in the intervention group.

The intervention consisted of 12 monthly emails, containing a total of 474 reminders about 415 patients. Each email contained a median of 7 reminders (IQR 2–24), with a range of 0–81 reminders in one email. The users notified us of 3 patients erroneously included in the reminders during the trial (false positive alerts). The source code can be downloaded at https://github.com/ace-dvm/BriefBot.

#### Primary outcome: Meta-analysis

**Fig 4:**
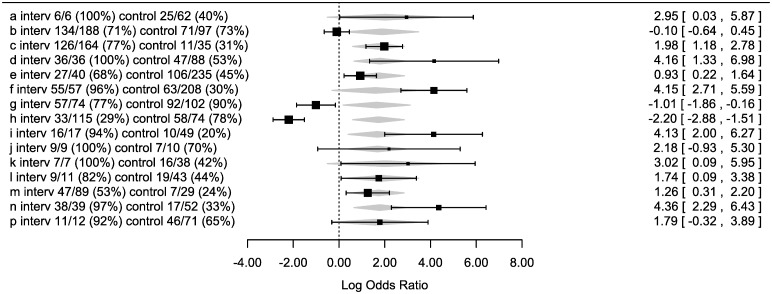
Forest plot of effect of intervention on each matched pair of clinicians, with correction for the (non-significant) differences in baseline performance (the percentage of patients with letters sent within 90 days of the visit) at the time of randomization. For each pair of doctors, we show the number of letters sent before 90 days/ the total number of letters which were due during the trial, and the resulting percentage of letters sent on time. The log odds ratio and confidence intervals are shown graphically (black bars = actual performance, grey diamonds = predicted performance according to the meta-analytic model), and numerically (in the right column).

The intervention group had significantly fewer overdue letters than the control group in the meta-analysis (OR = 5.7, CI = 1.9–17.2, p = 0.0020). The effect of the within-pair differences at baseline was not significant (p = 0.83). The results per pair are illustrated in the forest plot in Fig 4.

#### Secondary outcome: Analysis per patient

Analysis with the patient as the unit of analysis yielded similar results, with significantly fewer patients overdue for letters in the intervention than control group (OR = 4.4, CI = 1.5–12.4, p = 0.005).

#### Secondary outcome: Before-after

In the year of the trial, 598/4550 visits resulted in letters that were overdue by >90 days in the control group (13.1%, baseline = 9.5%), compared to 253/3140 in the intervention group (8.1%, baseline 9.1%). Compared to the period before the trial, there was a significant increase in overdue letters in the control group during the trial (**χ**^2^ = 0.31, p = < 0.0001). In the intervention group, overdue letters decreased non-significantly (**χ**^2^ = 2.17, p = 0.14).

#### Secondary outcome: Average time to write letters

For the control group, a median of 58 days (IQR 7.5–158.5) passed between the patient visit and the date that the associated letter was started (excluding letters that were not written). For the intervention group, this was significantly shorter at 30 days (IQR 6–82, p < 0.0001). Likewise, the median number of days elapsed between the visit date and the date the letter was actually sent was 77 in the control group (IQR 26–174) and 39 in the intervention group (IQR 12–88, p < 0.0001).

#### Sensitivity analysis: Number of patient visits per month

The relative workloads of the control vs intervention doctors, measured in terms of the (natural logarithm of) the ratio of the number of patients seen per month by the control/intervention doctor, did have a significant effect when added as a modifier to the meta-analytic model (OR = 2.61, CI = 1.1–6.1, p = 0.03). Thus, doctors who saw more patients wrote letters for a smaller percentage of their patients. However, the significance of the effect of the intervention persisted when corrected for the workload (OR = 3.8, CI = 1.4–10.7, p = 0.01).

#### Satisfaction

The survey consisted of 19 questions: 17 seven-point rating scale questions and 2 free text questions (see S1 File). The survey was sent to the twelve doctors who had received email reminders during the trial. All twelve were sent via email, and nine were also distributed in paper form. Seven responses were received, all using the paper form (58% of all doctors who received reminders during the trial, and 78% of doctors who were still working in the hospital at the time the survey was sent). Median overall satisfaction was rated as 6 of a possible 7 points (range 3 to 7), and six of the seven respondents indicated that they felt the system helped them write their letters on time (median score 6/7). The median score was 6 or better on use, usefulness, content, length, organization, value, and importance. Lower ratings (median score 4 or 5) were given on consistency, completeness, control of the content, age of information, and timing of delivery of the information (three indicated that the information came “too early” relative to when they need the information). No question had a median score lower than 4 (neutral). The free text comments were positive; three users indicated that they wanted the service to continue after the trial, and none indicated that they wanted it discontinued.

## Discussion

We found that a monthly email reminder was effective at improving the number and timeliness of letters sent to the GP by the hospital doctor from the outpatient clinic. The email contained a list of patients of the outpatient department who were (over)due for a letter to their GP. Doctors in the intervention group had 21% fewer patients without letters at 90 days after their visit, and they sent their letters a median of 48 days sooner than in the control group. Comparison with data from before the trial showed that the percentage of patients without letters had significantly increased in the control group and non-significantly decreased in the intervention group. User satisfaction with the system was very high, and doctors reported that they found the system useful and asked for it to be continued after the trial.

This study used a randomized controlled trial design and demonstrates that the intervention was effective on several outcome measures. We chose to employ a user-centered approach to designing the intervention, which probably contributed to its success. The system was extensively tested to minimize incorrect reminders, which also likely contributed to its acceptance. We used a meta-analytic approach to analyze the data, which can account for the clustered, paired randomization. We assessed the process-oriented outcomes of the intervention’s effect on writing letters, and also the effect of the system on its users through the use of a user satisfaction questionnaire. However, there are some limitations to this study. The control group had considerably more patient visits and patients due for letters than the intervention group. Although some of the difference between the groups seems to be attributable the difference in workload, the intervention still appeared to be effective after correction for this potential confounder. This was a single-center study with a large sample of patients but a fairly small number of participating doctors, and the variation between doctors at baseline was large. We accounted for this by using a matched-pair randomization, but our results should still be considered preliminary. We chose to use the number of letters sent by 90 days as the main outcome of the trial. Although the use of any threshold is a limitation, the threshold was chosen based on pre-trial data and the behavior of the system (the first reminder about a patient could arrive as late as 70 days after the visit). This and the positive effect demonstrated in the secondary outcomes make it unlikely that the choice of threshold affected our results. We chose to use the percentage of *all* patients who did not have a letter at 90 days after their visit as the outcome for pair matching and the before-after analysis, while we used only *patients who were due for a letter during the trial* to assess the outcome of the trial. We chose these outcomes because the first accounts for doctors who tend to write their letters before they are due, while the second more directly assesses the effect of reminders. Originally we planned to only include doctors who served in the outpatient department for at least 6 months, but due to circumstances such as maternity leave, this would have excluded 4 doctors and would have resulted in the need to extend the trial. Instead, we included doctors who had served on the outpatient department for at least 2 months, which was long enough to receive at least one reminder which included both new and recheck patients. Finally, although user satisfaction was extremely high, survey responses were anonymous, so we cannot assess for a response bias. However, 78% of users for whom we had current contact information responded, thus the response is at least representative of the majority of users.

An unexpected finding was that the before-after analysis showed only a slight, non-significant improvement in the primary outcome in the intervention group, and instead showed a significant decrease in performance in the control group. There are several possible explanations for this finding. One is that the hospital was in the process of moving from a partly paper-based system to an entirely electronic system (and, eventually, to a hospital-wide electronic patient record). In the interviews before the trial, the doctors reported that they would often leave the paper record of a patient on their desk as a reminder that a letter needed to be written. The move toward more electronic records may have disrupted this paper-based "reminder system." Another is that all participants were made aware of the intervention and were (necessarily) not blinded as to their assignment at randomization, and so control group doctors may have been demotivated by not getting support that was rather popular among their peers. During the trial the control arm doctors asked if the trial could be ended early so they could also receive the reminders, thus we have reason to believe that control-arm doctors may have felt they were at a disadvantage.

The software was written as open-source software. However, it needed to interface with hospital databases, and naturally the databases themselves cannot be provided alongside the source code. To provide a working demo, the database connection classes were replaced with classes which provide fictional patient data in the open source version. To use the software in another setting, these classes would need to be replaced with connections to the local database(s).

This study provides an example of an effective intervention that was non-interruptive, despite recent studies suggesting that interruptive interventions are more often effective [11,12,22]. However interruption can have unintended adverse consequences for patient care [13]. Thus finding effective, non-interruptive interventions is an important challenge for our profession. Factors which may have contributed to the success of the intervention are that the staff and residents use their hospital email account on a daily basis, which made it likely that doctors would see and read the emails from the system. The potential for doctors to use the reminders as a demonstrable measure of their performance may also have had a positive effect.

Prior work in this area includes other interventions directed at improving writing of discharge letters in a timely manner. These have involved conversion to computer-generated letters [23,24], or multi-faceted interventions of which automated reminders were a part [10,25]. To our knowledge, this is the first randomized trial investigating reminders for hospital doctors to write letters to the GP. Although this study took place in the Netherlands, throughout the world the patient letter is the typical means of communication between the hospital and GPs. Therefore, we expect that these findings would be directly applicable in other settings and countries, as well as indirectly applicable to other documentation and communication tasks.

Future work should investigate the barriers experienced by the doctors who responded less to the email reminders, and improve the service to meet their needs. For example, we hypothesize that emails which contain a long list of letters that need to be written may be discouraging, and that limiting the length of the email may improve acceptance. This intervention established the effect of a simple reminder on writing letters. The trial took place shortly before the implementation of an integrated electronic patient record system in our hospital, which should allow us to test whether additional features (such as providing a template letter) have an additional effect. Although this intervention used email reminders, in the more general terms suggested by the recently-published Two-Stream Model [26], it can be described as a system that combines an automated notification (in this case, the message title appearing in the inbox) with a user-controlled timing of delivery (opening the email to read the content). This general structure may be applicable in other contexts to address other clinical tasks which are important, but not urgent.

## Conclusions

A monthly email reminder was an effective intervention to improve the number and timeliness of letters written from the outpatient clinic to the general practitioner. Doctors’ satisfaction with the system was high, and they viewed it as a valuable service to assist them in providing good care for their patients. This study demonstrates that a non-interruptive intervention can be an effective form of clinical decision support.

## Supporting information

S1 File User satisfaction survey. The survey was formulated based on concepts from the Information Systems Success Model (DeLone/McLean model) and instantiated with questions based on the IBM Computer System Usability Questionnaire, the System Usability Scale, and the Computer User Satisfaction questionnaire.
(DOC)

S2 File R script for statistical analysis. (R)

S3 File Escalc file used by R script, calculated per patient-visit. (TAB)

S4 File Escalc file used by R script, calculated per patient. (TAB)

S5 File Data file used by R script, calculating the time between the visit and the letter. (TAB)

S6 File Data file used by R script, with the “before and after” results (before and during the trial). (TXT)

S7 File Spreadsheet containing count data, table calculating the number of patient-visits per doctor per month, and table calculating the number of letters needing to be written per doctor per month. (XLSX)
