# Supplementary material for: An email-based intervention to improve the number and timeliness of letters sent from the hospital outpatient clinic to the general practitioner: A pair-randomized controlled trial
Source: PLoS One. 2017 Oct 23;12(10):e0185812. doi: 10.1371/journal.pone.0185812 (PMC5653206; doi:10.1371/journal.pone.0185812)
Supplement: S1 File — The survey was formulated based on concepts from the Information Systems Success Model (DeLone/McLean model) and instantiated with questions based on the IBM Computer System Usability Questionnaire, the System Usability Scale, and the Computer User Satisfaction questionnaire. (DOC) [file pone.0185812.s001.doc]

Over the last year we have been conducting a trial of an email reminder system (“SnelleCor”) for letters to the *huisarts* for patients visiting the Polikliniek. Please let us know if you received the email reminders while you were seeing patients for Poli Interne, and let us know your opinions about this reminder system. Your individual responses will only be seen by the researcher and will be kept confidential. Your evaluation is important in understanding and improving workflow support. Thank you for helping us to improve.

**I received email reminders from Snelle Cor: Yes** **No**

→ If “No,” please skip to the last two questions.

| I read the emails | infrequently | 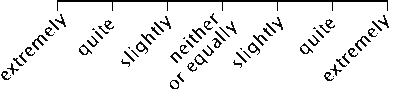 | frequently |
| --- | --- | --- | --- |
| I used the emails to find patients who needed letters | infrequently | 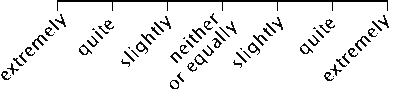 | frequently |
| I wrote letters sooner because of the emails | infrequently | 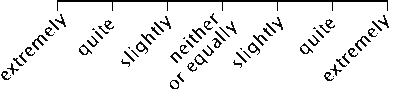 | frequently |
| The list of patients needing letters was | consistent | 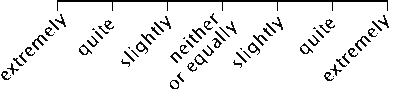 | inconsistent |
| The list of patients needing letters was | complete | 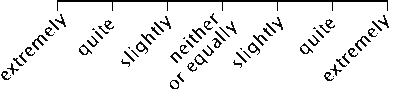 | incomplete |
| The “age” of the information at the time I read the emails was | reasonable | 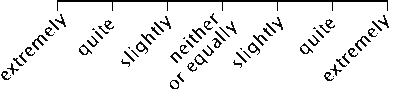 | unreasonable |
| The availability of output information at a time suitable for its use | late | 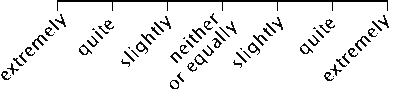 | early |
| The amount of information about each patient was | too little | 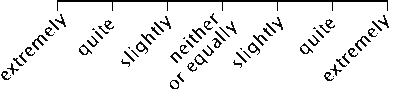 | too much |
| The length of the email was | long | 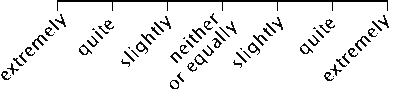 | short |
| The information in the emails was | useful | 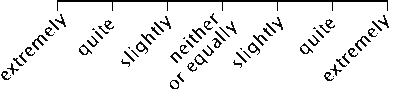 | useless |
| The organization of the information in the emails was | clear | 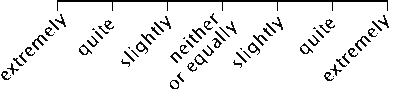 | confusing |
| For improving the timeliness of my letters and reducing the number of my patients without letters, the emails were | worthless | 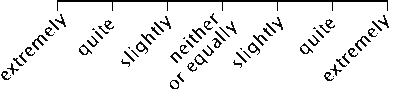 | valuable |
| For improving the timeliness of letters in the department and reducing the overall number of patients without letters, I think the emails were | worthless | 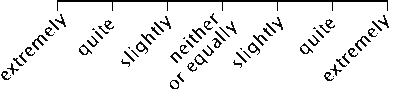 | valuable |
| My ability to control the content and delivery of the reminders (by replying to the emails) was | sufficient | 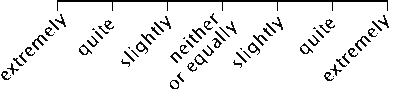 | insufficient |
| Generally, getting automatic reminders via email is | useful | 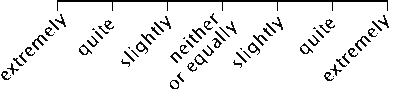 | useless |
| Generally, writing letters for the GP for poli patients is | unimportant | 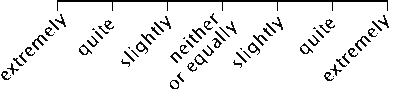 | important |
| Overall, my feeling about the emails is | satisfied | 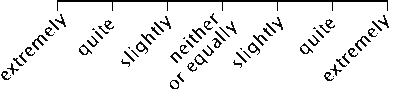 | unsatisfied |

**De volgende vragen mag u ook in Nederlands beantwoorden.**

Please give us your suggestions for how we can improve SnelleCor:

Please give us your suggestions for how we can improve the timeliness of letters to the GP:

Other comments:
